# Impairment of Mitochondrial Biogenesis and Dynamics Involved in Isoniazid-Induced Apoptosis of HepG2 Cells Was Alleviated by p38 MAPK Pathway

**DOI:** 10.3389/fphar.2017.00753

**Published:** 2017-10-26

**Authors:** Tianguang Zhang, Takashi Ikejima, Lizhong Li, Ruiqin Wu, Xiaoyan Yuan, Jun Zhao, Yimei Wang, Shuangqing Peng

**Affiliations:** ^1^Evaluation and Research Center for Toxicology, Institute of Disease Control and Prevention, PLA, Beijing, China; ^2^China-Japan Research Institute of Medical and Pharmaceutical Sciences, Shenyang Pharmaceutical University, Shenyang, China

**Keywords:** isoniazid, hepatotoxicity, apoptosis, mitochondrial biogenesis, mitochondrial fission, p38 MAPK

## Abstract

Isoniazid (INH), a widely used first-line antitubercular drug, has been noted to be associated with hepatotoxicity. In spite of extensive researches over many decades, the mechanism of INH-induced hepatotoxicity still remains poorly understood. Recently, mitochondrial toxicity has been emerging as a new paradigm for INH-induced hepatotoxicity. In this study, we showed that INH impaired mitochondrial biogenesis and dynamics in human hepatocarcinoma HepG2 cells. INH reduced mitochondrial membrane potential (MMP) and induced mitochondria swelling. INH also inhibited the protein expressions of three major mitochondrial biogenesis regulators, SIRT1, PGC1α and NRF1, along with increased acetylation of PGC1α. Meanwhile, INH decreased the number of mitochondria, accompanied by decreased expression of mitochondrial protein COX IV. INH caused mitochondrial fragmentation involving decreased levels of the fusion protein MFN2 as well as the fission protein DRP1. INH-reduced DRP1 expression was associated with the increase of apoptosis, suggesting the existence of pro-survival fission and its involvement in mitochondrial quality control. INH activated p38 MAPK, whereas inhibition of p38 MAPK aggravated INH-induced decreases of SIRT1, PGC1α, NRF1, COX IV and DRP1 expressions. P38 MAPK inhibition also further up-regulated the acetylation of PGC1α and exacerbated INH-induced MMP loss, mitochondrial swelling and apoptosis. Taken together, INH-activated p38 MAPK induced mitochondrial biogenesis to alleviate apoptosis through partly recovering SIRT1-PGC1α pathway activation. In the meantime, p38 MAPK activation by INH promoted protective mitochondrial fission to alleviate apoptosis by partial recovery of DRP1 expression.

## Introduction

Isoniazid is a widely used first-line agent for the treatment of tuberculosis. Despite the therapeutic benefits of this drug, it has been reported that INH mildly increased plasma alanine aminotransferase (ALT) levels up to approximately 20% in patients and that hepatotoxicity occurred in 1 of every 1,000 individuals treated with INH (Maddrey and Boitnott, 1973; Black et al., 1975; Nolan et al., 1999; Lee, 2003; Centers for Disease Control and Prevention [CDC], 2010). Worse yet, INH-induced hepatotoxicity has contributed to the emergence of multidrug-resistant strains of *Mycobacterium* tuberculosis, thereby limiting its application during the past decades (Tostmann et al., 2008). Multiple pathways are involved in INH-induced hepatotoxicity, such as oxidative stress, immune response and disruption of endogenous metabolism (Boelsterli and Lee, 2014). However, the classical paradigms for INH-induced hepatotoxicity have been changed over the past years (Zhai et al., 2008; Yue and Peng, 2009; Cheng et al., 2013). More recently, mitochondrial stress has been emerging as a new paradigm for INH-induced hepatotoxicity (Lee et al., 2013; Boelsterli and Lee, 2014).

Mitochondria are essential organelles for eukaryotic cells, being engaged in many important biological functions. Mitochondria have their own DNA and protein synthesizing system, which provide themselves with a capacity for autoreplication (Wallace, 2005). Mitochondrial biogenesis is a complex process, requiring the replication of mitochondrial DNA, large number of proteins encoded in the nucleus, and fewer ones derived from mitochondrial genes (Hock and Kralli, 2009). Peroxisome proliferator-activated receptor γ coactivator 1α (PGC1α) is a key regulator of mitochondrial biogenesis. It controls nucleus-mitochondria signaling pathways to stimulate mitochondrial biogenesis through interacting with its specific transcription factors (Fernandez-Marcos and Auwerx, 2011; Scarpulla et al., 2012). NRF1 is one that regulates many nucleus-encoded genes important for mitochondrial function and mass (Scarpulla, 2008).

PGC1α is regulated by mitogen-activated protein kinase p38 (p38 MAPK) and SIRT1. p38 MAPK is activated by Ca^2+^, cold, cytokines and exercise (Hock and Kralli, 2009; Fernandez-Marcos and Auwerx, 2011). SIRT1, a NAD^+^-dependent deacetylase, is activated by nutrient deprivation such as fasting and caloric restriction (Rodgers et al., 2008). p38 MAPK regulates PGC1α at the transcriptional and posttranslational levels, whereas SIRT1 acts on the posttranslational modification of PGC1α (Fernandez-Marcos and Auwerx, 2011). Furthermore, p38 MAPK regulates the stability of PGC1α (Puigserver et al., 2001). In addition to regulation of mitochondrial biogenesis, p38 MAPK and SIRT1 control mitochondrial function as well (Fan et al., 2004; Lagouge et al., 2006).

Mitochondria exist in dynamic networks undergoing fission and fusion (collectively termed mitochondrial dynamics) (van der Bliek et al., 2013). Mitochondrial morphologies change dramatically within cells by the opposing processes of fission and fusion. This dynamic behavior has important consequences for mitochondrial functions and participates in fundamental processes of cells, including development, apoptosis and aging (Chan, 2006). Mitochondrial fission and fusion were found to be mediated by multiple proteins, among which at least two dynamin-related GTPases, MFN2 and DRP1, play essential roles (Youle and van der Bliek, 2012). MFN2 is located in the outer mitochondrial membrane and mediates outer membrane fusion. DRP1 is involved in mitochondrial fission after its translocation from cytosol to mitochondria.

Mitochondrial toxicity is a major mechanism by which drugs cause liver injury (Labbe et al., 2008; Pessayre et al., 2010; Grattagliano et al., 2011). Recent works have revealed that INH induces mitochondrial dysfunction in liver, resulting in the occurrence of hepatotoxicity (Lee et al., 2013; Lee and Boelsterli, 2014). However, little is known about the roles of mitochondrial biogenesis and dynamics in INH-induced hepatotoxicity. In this study, we demonstrated that INH impaired mitochondrial biogenesis and dynamics, leading to apoptosis of human hepatocarcinoma HepG2 cells. INH simultaneously activated p38 MAPK to reduce mitochondrial biogenesis impairment against apoptosis by partly recovering the activation of SIRT1-PGC1α pathway. INH-activated p38 MAPK also induced mitochondrial fission against apoptosis by partial recovery of DRP1 expression.

## Materials and Methods

### Reagents and Antibodies

Isoniazid and SB203580 (SB) were obtained from Sigma-Aldrich (St. Louis, MO, United States). Mdivi-1, an inhibitor of DRP1, was purchased from Selleck Chemicals (Houston, TX, United States). Tetramethyl rhodamine methyl ester (TMRM), MitoTracker Deep Red FM, Hoechst 33342 and Lipofectamine RNAiMAX were obtained from Invitrogen (Grand Island, NY, United States). p38 MAPK-siRNA, a silencer negative control siRNA, and antibodies against p38 MAPK, phospho-p38 MAPK, NRF1, COX IV, cytochrome *c*, caspase 9, caspase 3, MFN2, DRP1, acetylated lysine, p-MAPKAPK-2, MAPKAPK-2 and β-actin were purchased from Cell Signaling Technology (Danvers, MA, United States). Antibodies against SIRT1 and Bax were obtained from Abcam (Abcam, Cambridge, United Kingdom). PGC1α antibody and protein A/G-agarose beads were obtained from Santa Cruz Biotechnology (Santa Cruz, CA, United States). All other chemicals were of analytical grade.

### Cell Culture

HepG2 cells, the human hepatocarcinoma cells, were originally obtained from ATCC. The cells were cultured in DMEM containing 10% FBS, 100 units/mL penicillin and 100 mg/mL streptomycin in a fully humidified incubator at 37°C with 5% CO_2_. The cells were routinely subcultured every 2–3 days.

### Detection of Mitochondrial Membrane Potential by Flow Cytometry

Mitochondrial membrane potential was determined using a fluorescent probe, TMRM. Cells were washed with phosphate buffered saline (PBS), and suspended in PBS containing TMRM at a concentration of 100 nM. After incubation for 30 min at 37°C, the fluorescent signals of TMRM were detected with a flow cytometer (FACSCalibur, BD Biosciences, San Jose, CA, United States) at 549 nm for excitation and 573 nm for emission.

### Western Blotting

Cells were washed twice with cold PBS and solubilized in RIPA lysis buffer (Applygen Technologies, Beijing, China) containing 50 mM Tris-HCl (pH 7.4), 150 mM NaCl, 1% NP-40, 0.1% SDS, protease inhibitor and protein phosphatase inhibitor (Applygen Technologies, Beijing, China) before use. After centrifugation for 15 min at 14,000 × *g*, protein concentration of the supernatant was quantified by bicinchoninic acid (BCA) protein assay kit (Applygen Technologies, Beijing, China). Equal amounts of protein (30–60 μg) were loaded for SDS-PAGE in the system of 12% separating gel and 5% stacking gel, then electrophoretically transferred to PVDF membranes and blocked with 5% no-fat milk in TBST buffer at room temperature for 2 h. Afterward, the membranes were incubated with the corresponding primary antibody overnight at 4°C and then incubated with appropriate horseradish peroxidase-conjugated secondary antibody. After the membranes were washed with TBST buffer, the proteins were detected with enhanced chemiluminescence detection reagents (Applygen Technologies, Beijing, China). The images were analyzed using Image J software (National Institutes of Health, Bethesda, MD, United States).

### Immunoprecipitation (IP)

HepG2 cells were lysed with IP lysis buffer. After centrifugation for 15 min at 14,000 × *g*, protein concentration of the supernatant was quantified by BCA protein assay kit. One milligram of protein was incubated with PGC1α antibody for 1 h at 4°C followed by incubation with protein A/G-agarose beads overnight. Beads were washed with IP lysis buffer for four times. Precipitants were subjected to western blotting/immunoblotting (IB) using acetylated lysine antibody and PGC1α antibody.

### Gene Silencing with siRNA

Cells were transfected with p38 MAPK-siRNA or a silencer negative control siRNA for p38 MAPK using Lipofectamine RNAiMAX according to the manufacturer’s instructions. After transfection, the cells were treated with INH for 24 h and then harvested. The inhibitory effect on p38 MAPK was evaluated with western blot analysis.

### Transmission Electron Microscopy

Cells were harvested and fixed with 3% glutaraldehyde in PBS for 2 h. After being rinsed three times with PBS before, the pellet was postfixed and stained with 1% osmium tetroxide and 1.5% potassium ferricyanide in PBS for 1 h. The pellet was then rinsed three times with water, and dehydrated in increasing concentrations of ethanol (30, 50, 70, 85, and 95%) for 15 min, followed by dehydration in 100% anhydrous ethanol for three times for 20 min each time. After dehydration, the pellet was infiltrated with a series of increasing concentrations of Spurr’s resin in ethanol, consisting of 25, 50, 75, and 100% in dry acetone and subsequently polymerized at 60°C for 24 h. Embedded cells in resin blocks were cut to ultrathin sections (70 nm) using a Leica UC6 ultramicrotome (Leica Microsystems, Wetzlar, Germany), stained with uranyl acetate and lead citrate, and viewed with a transmission electron microscope (Hitachi H-7650, Hitachi, Tokyo, Japan).

### Confocal Microscopy

Cells were pre-stained with MitoTracker Deep Red FM (MitoTracker) for 30 min at 37°C followed by fixation in 4% paraformaldehyde for 20 min at room temperature. After being washed with PBS, the cells were then permeabilized with 0.1% Triton X-100 in PBS and blocked at room temperature with PBS containing 0.1%Triton X-100 and 5% normal goat serum. The treated cells were incubated with primary antibodies at 4°C overnight and then incubated with Anti-mouse IgG Alexa Fluor^®^ 488 Conjugate to probe Bax and Anti-rabbit IgG Alexa Fluor^®^ 488 Conjugate to probe cytochrome *c* for 1 h at room temperature. After washing in PBS, Hoechst 33342 was used to stain the nucleus. Fluorescent images were visualized using a confocal microscope (Zeiss LSM880, Carl Zeiss, Jena, Germany). Colocalization analysis was performed by intensive correlation analysis (ICA) method of WCIF ImageJ software (Wright Cell Imaging Facility).

### Statistical Analysis

Quantitative data were presented as the means ± SD of at least triplicates. Statistical differences among individual groups were determined using one-way ANOVA. All analyses were done using SPSS 18.0 statistical software. A value of *P* < 0.05 was considered statistically significant.

## Results

### INH Inhibited Mitochondrial Biogenesis

**FIGURE 1:**
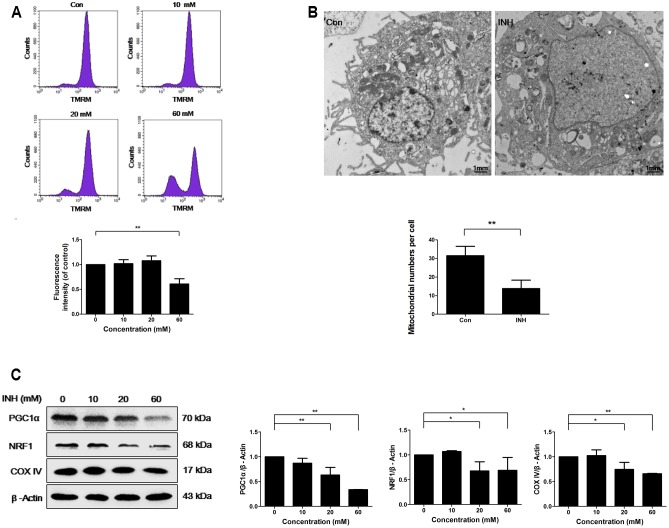
INH inhibited mitochondrial biogenesis. **(A)** HepG2 cells were exposed to the indicated concentrations of INH for 24 h, stained with TMRM, then harvested for measurement of mitochondrial membrane potential (MMP) by flow cytometry. The lower parts of **(A)**, geometric mean (Geo Mean) was used to calculate the intensity of fluorescence. **(B)** Transmission electron microscopy was performed on HepG2 cells to examine the alteration in mitochondrial mass. Ten micrographic fields were randomly selected in each group. **(C)** Detection of protein expressions of PGC1α, NRF1 and COX IV by western blotting after treatment with different concentrations of INH for 24 h. Right parts of **(C)**, PGC1α, NRF1 and COX IV quantitated by densitometry and plotted after normalization to β-actin. Results are the representatives of at least three independent experiments, respectively. Data are presented as mean ± SD of at least three independent experiments. ^∗^*P* < 0.05, ^∗∗^*P* < 0.01.

INH treatment for 24 h induced concentration-dependent loss of MMP in HepG2 cells, indicating the impairment of functional integrity of mitochondria by INH (**Figure 1A**).

To examine whether INH was able to influence mitochondria mass, transmission electron microscopy (TEM) was used. TEM detection showed that INH decreased mitochondrial density in HepG2 cells (**Figure 1B**). We next detected the expression of mitochondria-specific protein COX IV. As shown in **Figure 1C**, COX IV expression was concentration-dependently decreased in INH-treated cells at 24 h.

PGC1α and NRF1 are two essential regulating factors for mitochondrial biogenesis (Dominy and Puigserver, 2013). To determine whether the decrease in mitochondria mass resulted from impaired biogenesis, the protein expressions of PGC1α and NRF1 were evaluated. As shown in **Figure 1C**, PGC1α and NRF1 expressions were decreased upon INH treatment concentration-dependently.

These results suggested that INH impaired mitochondrial biogenesis by the inhibition of PGC1α pathway.

### INH Activated p38 MAPK That Regulated Mitochondrial Biogenesis

**FIGURE 2:**
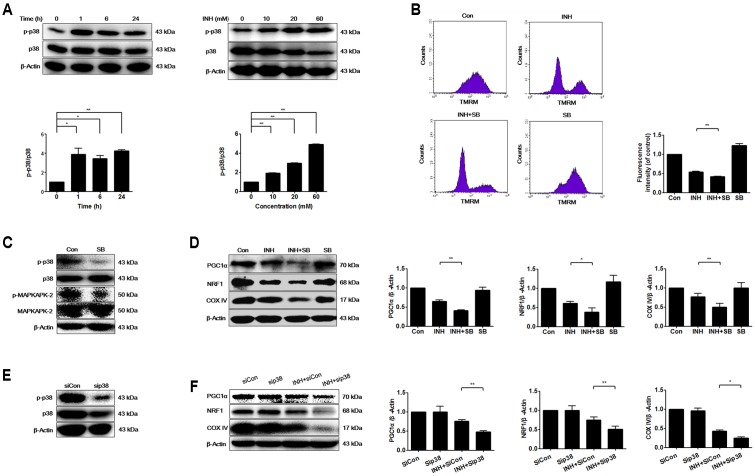
INH activated p38 MAPK that regulated mitochondrial biogenesis. **(A)** Protein expressions of phosphorylated p38 MAPK (p-p38) and p38 MAPK were detected by western blotting after treatment of HepG2 cells with indicated concentrations of INH for 24 h, or with INH (60 mM) for indicated time. Lower parts of **(A)** quantitated by densitometry and plotted after normalization with p38. HepG2 cells were exposed to INH (60 mM) for 24 h in the presence or absence of p38 inhibitor SB (20 μM), MMP was determined by flow cytometry **(B)**, and expressions of p-p38 MAPK, p38 MAPK, p-MAPKAPK-2 and MAPKAPK-2 **(C)** as well as PGC1α, NRF1 and COX IV were analyzed by western blotting **(D)**. Right parts of **(B)**, geometric mean (Geo Mean) was used to calculate the intensity of fluorescence. Right parts of **(D)**, the expressions of PGC1α, NRF1 and COX IV are quantitated by densitometry and plotted after normalization with β-actin. HepG2 cells were transfected with Silencer Negative Control siRNA (siCon) or p38 MAPK-siRNA (sip38), and the expressions of p38 MAPK was verified by western blotting **(E)**, as well as the expressions of PGC1α, NRF1 and COX IV were analyzed by western blotting **(F)**. Right parts of **(F)** the expressions of PGC1α, NRF1 and COX IV are quantitated by densitometry and plotted after normalization with β-actin. Results are the representatives of at least three independent experiments, respectively, and data are presented as mean ± SD of at least three independent experiments. ^∗^*P* < 0.05, ^∗∗^*P* < 0.01.

Mitochondrial biogenesis may be regulated by p38 MAPK (Wenz, 2013). As presented in **Figure 2A**, INH activated p38 MAPK in time- and dose-dependent manners. To investigate whether p38 MAPK played a role in mitochondrial biogenesis, we tested what inhibition of p38 MAPK resulted in. Compared with INH treatment alone, INH together with p38 MAPK inhibitor SB (**Figure 2C**) markedly decreased MMP (**Figure 2B**) and significantly decreased the protein expressions of PGC1α and NRF1 (**Figure 2D**). In agreement with the down-regulated PGC1α and NRF1 expressions, COX IV expression was also further decreased following p38 MAPK inhibition (**Figure 2D**). To eliminate the potential non-specific effects of SB, we partially knocked down p38 MAPK by siRNA transfection against p38 MAPK in HepG2 cells (**Figure 2E**). Consistently, siRNA-mediated knockdown of p38 MAPK diminished the protein expressions of PGC1α, NRF1, and COX IV (**Figure 2F**).

These data indicated that INH-activated p38 MAPK alleviated the impairment of mitochondrial biogenesis induced by INH.

### p38 MAPK Regulated SIRT1 to Reduce INH-Mediated Impairment of Mitochondrial Biogenesis

**FIGURE 3:**
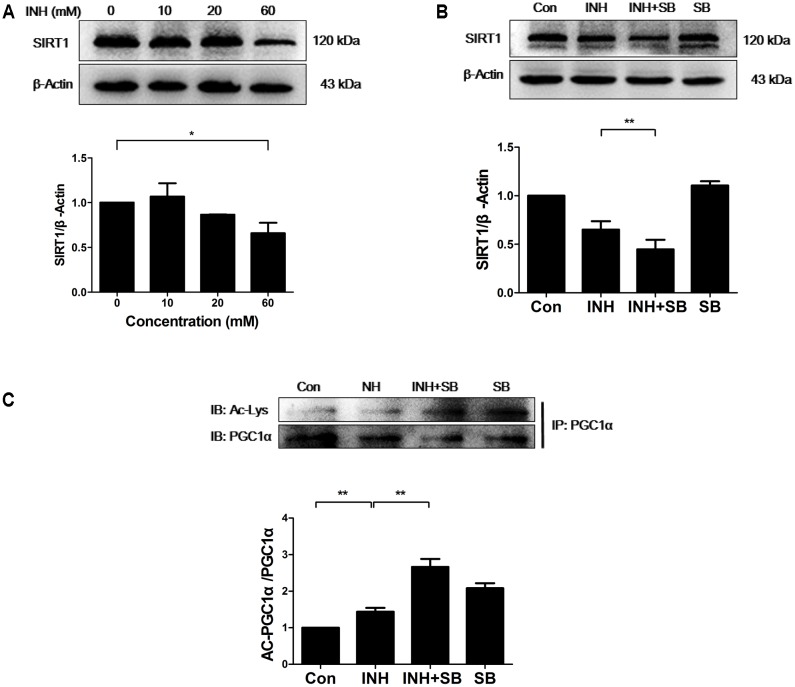
p38 MAPK regulated SIRT1 to reduce INH-mediated impairment of mitochondrial biogenesis. **(A)** Detection of SIRT1 expression by western blotting after treatment with indicated concentrations of INH for 24 h. **(B)** HepG2 cells were exposed to INH (60 mM) for 24 h in the presence or absence of a p38 MAPK inhibitor SB (20 μM) and the expression of SIRT1 was analyzed by western blotting. **(C)** HepG2 cells treated with INH (60 mM) for 24 h in the presence or absence of SB were harvested and subjected to immunoprecipitation using anti-PGC1α antibody, and western blotting were performed for the indicated proteins. The upper parts of **(A–C)** indicate the representative blots of at least three independent experiments, the lower parts indicate the corresponding results of densitometric analyses of blots after normalization to β-actin. Data are presented as mean ± SD of at least three independent experiments. Ac-Lys, acetylated lysine; AC-PGC1α, acetylated PGC1α, ^∗^*P* < 0.05, ^∗∗^*P* < 0.01.

It is well known that SIRT1 promotes mitochondrial biogenesis through the activation of PGC1α by deacetylation (Chalkiadaki and Guarente, 2012). Therefore, SIRT1 protein expression was determined upon INH treatment. As expected, INH inhibited SIRT1 expression (**Figure 3A**), indicating the increase of SIRT1 acetylation and subsequent inactivation of PGC1α. As shown in **Figure 3B**, INH-induced decrease of SIRT1 expression was exacerbated by p38 inhibitor SB. At the same time, acetylation of PGC-1α was also further increased by SB (**Figure 3C**), suggesting the further decrease in PGC1α activity.

Together, these data indicated that INH-activated p38 MAPK partially recovered SIRT1-PGC1α pathway, thereby reducing impaired mitochondrial biogenesis.

### p38 MAPK-Independent Fusion Mechanism Was Involved in INH-Induced Mitochondrial Fragmentation

**FIGURE 4:**
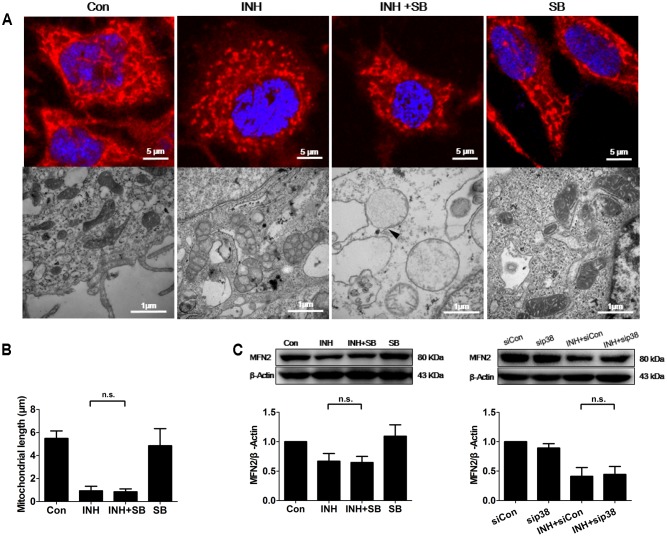
p38 MAPK-independent fusion mechanism was involved in INH-induced mitochondrial fragmentation. **(A)** INH-introduced mitochondrial fission and ultrastructural changes in mitochondria. Upper row: mitochondria stained with MitoTracker deep red; lower row: ultrastructural changes in mitochondria detected by TEM. **(B)** Quantatition of the length of mitochondria stained with MitoTracker deep red in **(A)**. Twenty cells from 4 micrographic fields were randomly selected in each group. **(C)** Detection of MFN2 expression levels by western blotting after inhibition of p38 MAPK by p38 MAPK inhibitor SB or p38 MAPK-siRNA (sip38). Results are the representatives of at least three independent experiments, respectively, and data are presented as mean ± SD of at least three independent experiments. Silencer Negative Control siRNA, siCon; n.s., not significant; Arrowhead: broken mitochondrial membrane.

INH induces apoptosis of HepG2 cells (Schwab and Tuschl, 2003; Bhadauria et al., 2010). Mitochondria usually undergo extensive fragmentation during apoptosis (Youle and Karbowski, 2005). Long, tubular, and branching mitochondria spreading throughout the cytoplasm were observed in control cells. In contrast, INH-treated cells presented small, round mitochondria, lacking of the branching seen in controls (**Figure 4A**). Reduction of mitochondrial length was found to the bottom by INH treatment alone, where inhibition of p38 MAPK expression could not contribute to (**Figures 4A,B**).

TEM and morphometric analysis of mitochondrial damage also revealed extensive alterations in mitochondrial ultrastructure (**Figure 4A**). In control group, mitochondria appeared primarily elongated morphology, normal cristae and high-density matrix. Conversely, INH caused mitochondrial swelling, reduction and defects in cristae, and reduction in matrix density. Inhibition of p38 MAPK exacerbated INH-induced abnormal appearance of mitochondria, including severe swelling, vanished cristae, lower-density matrix and broken mitochondrial membrane (arrowhead).

Mitochondrial fragmentation could be regulated by the fusion protein MFN2. Consistent with the results in **Figures 4A,B**, INH decreased the expression of MFN2 to the bottom where blockade of p38 MAPK with either a chemical inhibitor or siRNA could not contribute further more (**Figure 4C**).

These results suggested that INH alone decreased MFN2 to the level where p38 MAPK was not associated with, resulting in mitochondrial fragmentation.

### p38 MAPK Regulated DRP1 to Promote INH-Induced Mitochondrial Fragmentation against Apoptosis

**FIGURE 5:**
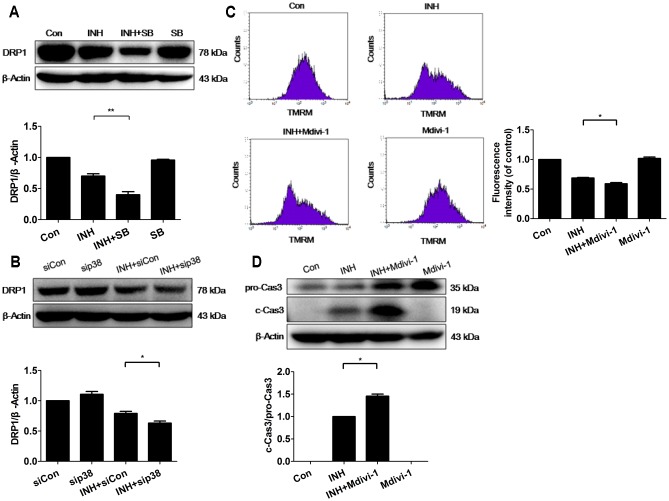
p38 MAPK regulated DRP1 to promote INH-induced mitochondrial fragmentation against apoptosis. **(A)** Detection of DRP1 expression by western blotting after inhibition of p38 MAPK by p38 MAPK inhibitor SB. **(B)** Detection of DRP1 expression by western blotting after inhibition of p38 MAPK by p38 MAPK-siRNA (sip38). HepG2 cells were exposed to INH (60 mM) for 24 h in the presence or absence of mitochondrial DRP1 inhibitor Midivi-1 (5 μM) and MMP was determined by flow cytometry **(C)**, and the expression of cleaved caspase 3 was analyzed by western blotting **(D)**. The upper parts of **(A,B,D)** indicate the representative blots of at least three independent experiments; the lower parts indicate the corresponding results of densitometric analyses of blots. Right parts of **(C)**, geometric mean (Geo Mean) was used to calculate the intensity of fluorescence and data are presented as mean ± SD of at least three independent experiments. Silencer Negative Control siRNA, siCon; ^∗^*P* < 0.05, ^∗∗^*P* < 0.01.

Fission mechanism was examined in INH-introduced mitochondrial fragmentation. Unexpectedly, INH inhibited the expression of DRP1 (**Figure 5A**). Moreover, INH further reduced DRP1 expression after blockade of p38 MAPK with either a chemical inhibitor or siRNA (**Figures 5A,B**). The expression of DRP1 was reduced, but the possibility that INH-induced mitochondrial fragmentation partly relied on DRP1 cannot be completely excluded.

To identify the role of DRP1 in INH-induced mitochondrial dysfunction, we evaluated the alterations of MMP with or without DRP1 inhibitor Mdivi-1. As presented in **Figure 5C**, Mdivi-1 exacerbated INH-induced loss of MMP in HepG2 cells.

To identify the role of DRP1 in INH-induced apoptosis, we evaluated the effect of Mdivi-1 on the cytotoxicity induced by INH. Compared with INH treatment alone, INH together with Mdivi-1 significantly enhanced apoptosis induction, evidenced by elevated cleavage of apoptotic protein of procaspase 3 (**Figure 5D**).

These results suggested that mitochondrial fission caused by DRP1 might contribute to the elimination of impaired mitochondria, and that p38 MAPK worked for the protection from apoptosis by up-regulation of DRP1.

### p38 MAPK Inhibited INH-Induced Bax Translocation to Mitochondria

**FIGURE 6:**
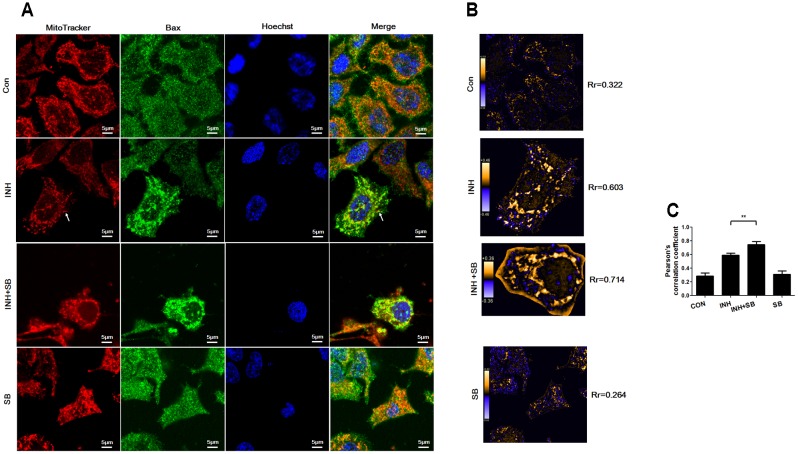
p38 MAPK regulated INH-induced Bax translocation to mitochondria. **(A)** Colocalization of Bax with mitochondria. Cells were treated with INH (60 mM) for 24 h with or without SB and probed with MitoTracker deep red, primary anti-Bax antibody /secondary Anti-mouse IgG Alexa Fluor^®^ 488 Conjugate and then Hoechst 33342. Yellow dots in the merged figures indicated active Bax (green) on the mitochondria (red) location. The nuclei were blue. **(B)** Qualitative analysis of colocalization by PDM images. The PDM images were pseudocolored, where each pixel is equal to the PDM value at that location and a PDM scale bar was inserted. The orange color indicates colocalized pixels and the blue color suggests segregation. The PDM value is the product of the differences from the mean. For each pixel: PDM = (red intensity – mean red intensity) × (green intensity – mean green intensity). Rr, Pearson’s correlation coefficient. **(C)** Analysis of pearson’s correlation coefficient. Twenty cells from 4 micrographic fields were randomly selected in each group. Arrowheads: the elongated tubular mitochondria located with Bax. Data are presented as mean ± SD of at least three independent experiments. ^∗∗^*P* < 0.01.

During apoptosis, Bax recruitment to mitochondria frequently occurs during mitochondrial fragmentation (Martinou and Youle, 2011). As shown in **Figure 6A**, Bax (green) shows diffuse cytoplasmic localization with a minor fraction attached to mitochondria (red) in untreated cells (showing yellow dots in the merged figure). For the cells treated with INH, focal clusters of Bax localized to mitochondria and the colocalization was increased after inhibition of p38 MAPK with SB (**Figure 6A**). PDM images also indicated INH induced obviously overlapped pixels shown as orange pixels which were further augmented after inhibition of p38 MAPK, along with the increases in the pearson’s correlation coefficient (Rr) (**Figures 6B,C**). Bax clusters not only located at mitochondrial fragments but also presented at elongated tubular mitochondria (**Figure 6A**, arrowheads).

Our results indicated that p38 MAPK partially inhibited INH-induced Bax translocation to mitochondria.

### p38 MAPK Inhibited INH-Induced Cytochrome *c* Release

**FIGURE 7:**
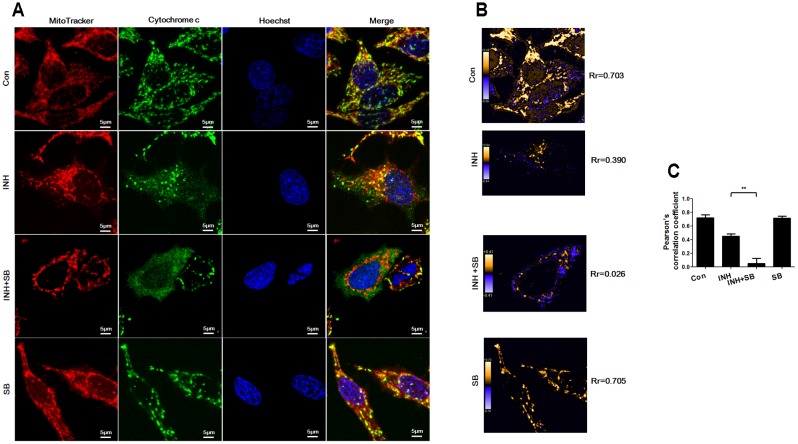
p38 MAPK regulated INH-induced cytochrome *c* release. HepG2 cells were treated with INH (60 mM) for 24 h with or without SB and probed with MitoTracker deep red, primary anti-cytochrome *c* antibody/secondary Anti-rabbit IgG Alexa Fluor^®^ 488 Conjugate and then Hoechst 33342. **(A)** Yellow parts in the merged figures indicate cytochrome *c* (green) resided in the mitochondria (red). **(B)** Qualitative analysis of colocalization by PDM images. Rr, Pearson’s correlation coefficient. **(C)** Analysis of pearson’s correlation coefficient. Twenty cells from 4 micrographic fields were randomly selected in each group. Data are presented as mean ± SD of at least three independent experiments. ^∗∗^*P* < 0.01.

After translocation to mitochondria, Bax permeabilizes mitochondrial outer membrane to allow efflux of cytochrome *c* (Martinou and Youle, 2011). As shown in **Figure 7A**, cytochrome *c* (green) was localized in mitochondria (red) in control group (yellow in the merged figures). In contrast, INH caused most cytochrome *c* release to cytoplasm with only a few retained in fragmented mitochondria (yellow dots in the merged figures). In the presence of p38 MAPK inhibitor SB, cytochrome *c* was almost completely released from mitochondria into cytoplasm accompanied by mitochondrial fragmentation. PDM images also indicated overlapped pixels were further reduced after inhibition of p38 MAPK, along with the decreases in the Rr (**Figures 7B,C**).

Our data suggested that p38 MAPK activation prevented INH-induced cytochrome *c* release.

### p38 MAPK-Knockdown Exacerbated INH-Induced Apoptosis

**FIGURE 8:**
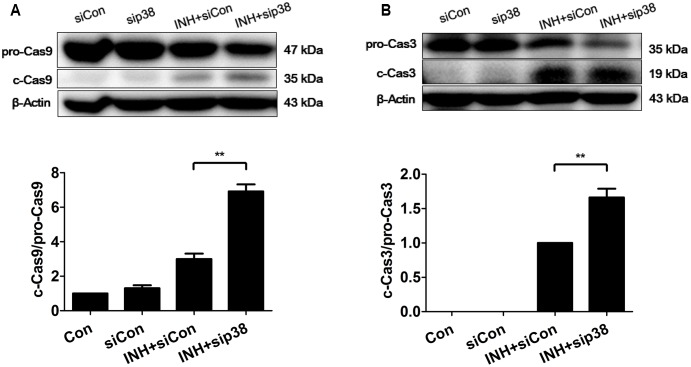
p38 MAPK knockdown exacerbated INH-induced apoptosis. HepG2 cells were exposed to INH (60 mM) for 24 h after transfection with Silencer Negative Control siRNA (siCon) or p38 MAPK-siRNA (sip38), and the cleavages of caspase 9 **(A)** and caspase 3 **(B)** were detected by western blotting. Results are the representatives of at least three independent experiments, the upper parts of **(A,B)** indicate the representative blots of at least three independent experiments, respectively; the lower parts indicate the corresponding results of densitometric analyses of blots. Data are presented as mean ± SD of at least three independent experiments. procaspase 9, pro-Casp9; procaspase 3, pro-Casp3; cleaved caspase 9, c-Cas9; cleaved caspase 3, c-Cas3; ^∗∗^*P* < 0.01.

In line with the results of confocal microscopy, down-regulation of p38 MAPK by siRNA transfection exacerbated intrinsic (mitochondrial) pathway-mediated apoptosis in INH-treated cells through the activations of caspases 9 and 3 (**Figure 8**).

## Discussion

Mitochondrial quality control or dynamic stability is maintained by biogenesis and degradation, as well as by balanced antagonistic dynamic processes of fusion (regeneration of damage) and fission (elimination of damage) (Westermann, 2010, 2012; Youle and van der Bliek, 2012; Archer, 2013; van der Bliek et al., 2013). When mitochondria become unstable due to various stresses, mitochondrial biogenesis and dynamics contribute to mitochondrial homeostasis. The present report demonstrated that INH impaired mitochondrial biogenesis and dynamics. The finding that inhibition of p38 MAPK aggravated this situation implicated that simultaneous activation of p38 MAPK by INH alleviated INH-induced mitochondrial unstability. INH may disturb the mitochondrial homeostasis through repressing mitochondrial biogenesis as well as disturbing mitochondrial dynamic stability, leading to eventual apoptosis.

Mitochondria, the main energy source in hepatocytes, play a key role in metabolism and homeostasis of liver (Grattagliano et al., 2011). It is therefore not surprising that mitochondria function as a center of signaling pathways mediating hepatocyte injury (Pessayre et al., 2012). Mitochondrial biogenesis is a tightly regulated process requiring the coordination of two genomes between nucleus and mitochondria (Ryan and Hoogenraad, 2007). PGC1α, an important transcription factor coactivator, promotes the transcription of NRF1 by binding to NRF1 to drive actually all aspects of mitochondrial biogenesis, including activating transcription of fatty acid oxidation and respiratory chain genes, enhancing mitochondrial respiratory capacity, and increasing mitochondrial mass (Scarpulla et al., 2012). Our results showed that INH decreased mitochondrial mass by reducing the expressions of PGC1α and NRF1, accompanied by the loss of MMP. These suggested that INH inhibited mitochondrial biogenesis leading to energy metabolism disorder in HepG2 cells.

p38 MAPK regulates mitochondrial biogenesis by stimulation of PGC1α. p38 MAPK plays a stimulatory role in PGC1α transcription in mouse primary hepatocytes (Cao et al., 2005). However, p38 MAPK was also involved in the negative regulation of mitochondrial biogenesis. It was reported that p38 MAPK activation decreased PGC1α expression in myotubes exposed to palmitate leads (Crunkhorn et al., 2007), In addition, the cardiac mitochondria from transgenic mice overexpressing the p38 MAPK activator MAPK-kinase-6 (MKK6) showed lower oxidative respiration (Wall et al., 2006). Therefore, p38 MAPK can both positively and negatively regulate mitochondrial biogenesis depending on cellular context. Here we showed INH-activated p38 MAPK alleviated mitochondrial biogenesis impairment, suggesting p38 MAPK positively regulated mitochondrial biogenesis in HepG2 cells under INH treatment. Moreover, PGC1α can enhance the expression of NRF1 (Brenmoehl and Hoeflich, 2013). Therefore, the finding that p38 MAPK inhibition exacerbated INH-induced decrease of NRF1 expression, can be accounted for the down-regulation of PGC1α. Taken together, p38 MAPK could promote two mitochondrial biogenesis regulators PGC1α and NRF1 to stabilize mitochondrial mass.

SIRT1 is also able to promote mitochondrial biogenesis through deacetylation and activation of PGC1 (Rodgers et al., 2005; Gerhart-Hines et al., 2007), resulting in the up-regulations of numerous genes that encode proteins for mitochondrial biogenesis, energy production and oxidative phosphorylation (Rodgers et al., 2008; Brenmoehl and Hoeflich, 2013). In healthy human volunteers, a novel SIRT1 activator SRT2104 increased the recovery of adenosine diphosphate and phosphocreatine and decreased serum lipid levels after exercise, suggesting an increase in mitochondrial oxidative phosphorylation (Libri et al., 2012). Hepatocyte-specific deletion of SIRT1 resulted in hepatic steatosis and inflammation through changing fatty acid metabolism (Purushotham et al., 2009). Furthermore, previous studies showed that modest overexpression of SIRT1 led to a protective effect on high-fat induced glucose intolerance and hepatic steatosis (Banks et al., 2008; Pfluger et al., 2008). Therefore, SIRT1 also plays a vital role in maintaining normal liver functions. The finding that SIRT1 suppression by INH was consistent with the decrease of PGC1α expression and increase of PGC-1α acetylation, suggesting INH regulated PGC1α through both transcriptional and posttranslational modifications. Such double regulations of protein expression and protein activity enabled INH to suppress PGC1α function strongly. In addition, inhibition of p38 MAPK exacerbated INH-induced decrease of SIRT1, suggesting that p38 MAPK is an up-regulator of SIRT1. Accordingly, p38 MAPK also indirectly activated PGC1α by the activation of SIRT1 to partly recover mitochondrial biogenesis. Overall, p38 MAPK not only directly exerted a stimulatory effect on PGC1α expression, but also indirectly activated PGC1α by stimulating SIRT1 to reduced INH-induced mitochondrial biogenesis impairment.

Mitochondria are crucial for apoptosis progression through the release of cytochrome *c*, when mitochondrial outer membrane permeabilization (MOMP) occurs due to the actions of pro-apoptotic Bcl-2 family members Bax and/or Bak (Chan, 2012). Our results indicated that INH activated intrinsic (mitochondrial) apoptotic pathway, including the activation of Bax, release of cytochrome *c* and loss of MMP. These suggested that INH not only inhibited the supplement of functional mitochondria by impairing mitochondrial biogenesis, but also induced dysfunction of existing mitochondria, both of which resulted in mitochondrial toxicity. Inhibition of p38 MAPK exacerbated this mitochondrial toxicity and therefore apoptosis. These suggested that p38 MAPK activation had a protective effect against INH-induced apoptosis through maintaining mitochondrial biogenesis and function.

While fragmentation of mitochondria is a common phenomenon during apoptosis (Youle and Karbowski, 2005; Westermann, 2010; Martinou and Youle, 2011), whether mitochondrial fragmentation is crucial for apoptosis progression is under dispute. Initial researches suggested that inhibition of mitochondrial fragmentation by ablation of DRP1 reduced cytochrome *c* release and apoptosis (Breckenridge et al., 2003; Germain et al., 2005; Brooks et al., 2007). Conversely, other studies showed that inhibition of DRP1 partially reduced (Ishihara et al., 2009) or had little effect (Wakabayashi et al., 2009) on cytochrome *c* release, without affecting apoptosis (Wakabayashi et al., 2009). Increases in apoptotic neurons were induced in the embryonic and adult brains of DRP1 mutant mice (Ishihara et al., 2009). Inhibiting mitochondrial fragmentation through enforced fusion obtained similar results. Overexpression of MFN2 reduced cytochrome *c* release and apoptosis (Jahani-Asl et al., 2007), while in other study, overexpression of MFN1, MFN2 or Opa1 had no effect on cytochrome *c* release or apoptosis (Sheridan et al., 2008). We showed here that inhibition of DRP1 with Mdivi-1 could aggravate apoptosis under INH treatment. Because mitochondrial quality control involved rescue of damage by fusion and elimination of damage by fission (Westermann, 2010; Youle and van der Bliek, 2012; van der Bliek et al., 2013), it was possible that INH introduced mitochondrial toxicity beyond a certain threshold so that rescue of damage by fusion was impaired, whereas mitochondrial fragmentation contributed to maintenance of mitochondrial health by segregating the seriously damaged portion of mitochondria. In addition, mitochondrial fragmentation is also important for mitochondrial inheritance during cell division (Westermann, 2010). Therefore, INH-induced mitochondrial fragmentation can compensate for the decreased mitochondrial number concomitant with the elimination of damaged mitochondria. Together, mitochondrial fragmentation can counteract INH-induced apoptosis by alleviating mitochondrial damage through two synergistic processes: compensation of functional mitochondria and elimination of dysfunctional mitochondria.

Mitochondrial fragmentation can be caused by enhanced fission, reduced fusion, or both (Youle and van der Bliek, 2012; Dominy and Puigserver, 2013; van der Bliek et al., 2013). In the present study, interesting results were that INH induced mitochondrial fragmentation with decreased expression of MFN2, but DRP1 expression was also inhibited. Compared with INH treatment alone, INH together with p38 MAPK inhibition further decreased the protein level of DRP1 without affecting MFN2 expression and the shortened mitochondrial length. These indicated that INH seemingly reduced the pro-survival mitochondrial fission by inhibiting fission mediator DRP1. But in fact, cells finally maintain cytoprotective mitochondrial fragmentation by compensatory down-regulation of fusion mediator MFN2 to keep mitochondrial health. Inhibition of p38 MAPK seemingly had no effect on the shortened mitochondrial length in INH-treated HepG2 cells, since p38 MAPK inhibition not only exacerbated INH-induced decrease in mitochondrial fission activity, but also restricted further compensatory decrease in fusion activity. This synergistic effect finally suppressed the shortened mitochondria to continuously fragmentate into shorter ones. Due to the deficiency in elimination of damage by mitochondrial fission, INH together with p38 MAPK inhibition simultaneously aggravated INH-caused mitochondrial toxicity, evidenced by lower level of MMP and more severe mitochondrial swelling. Overall, INH induced pro-survival mitochondrial fragmentation mainly through the decrease of MFN2 expression, nevertheless, the reduced DRP1 expression also at least partly contributed to mitochondrial fragmentation. Meanwhile, p38 MAPK promoted mitochondrial fission to maintain mitochondrial health by partial recovery of DRP1 expression. Consequently, p38 MAPK counteracted INH-induced mitochondrial toxicity to allow cells to escape from apoptosis through two synergistic mechanisms: supplement of energy demands by mitochondrial biogenesis and compensation of damage by mitochondrial fission.

A direct connection between the mitochondrial fission and apoptosis was obtained by studying the mitochondrial localization of Bax. During apoptosis, mitochondrial morphology changes, fragmentating into small and round parts near the time of Bax activation and cytochrome *c* release (Youle and van der Bliek, 2012). Bax has been shown to colocalize with DRP1 and MFN2 during apoptosis (Karbowski et al., 2002). When DRP1 expression was inhibited using RNAi, the induction of apoptosis was also inhibited (Youle and Karbowski, 2005). In addition, silencing of MFN2 increased cell sensitivity to apoptotic stimuli (Neuspiel et al., 2005; Shen et al., 2007). These directly linked the localization of Bax with the fragmentated mitochondria when HepG2 cells underwent apoptosis. Fragmentated mitochondria caused by INH were always connected with Bax accompanied by down-regulations of MFN2 and DRP1, but we noticed that some Bax clusters were located at the elongated tubular mitochondria without complete fragmentation into small parts. These results indicated that INH caused mitochondrial fragmentation to counteract apoptosis, possibly by a mechanism of recruitment of Bax to the mitochondria and subsequent interactions with MFN2 and/or DRP1.

**FIGURE 9:**
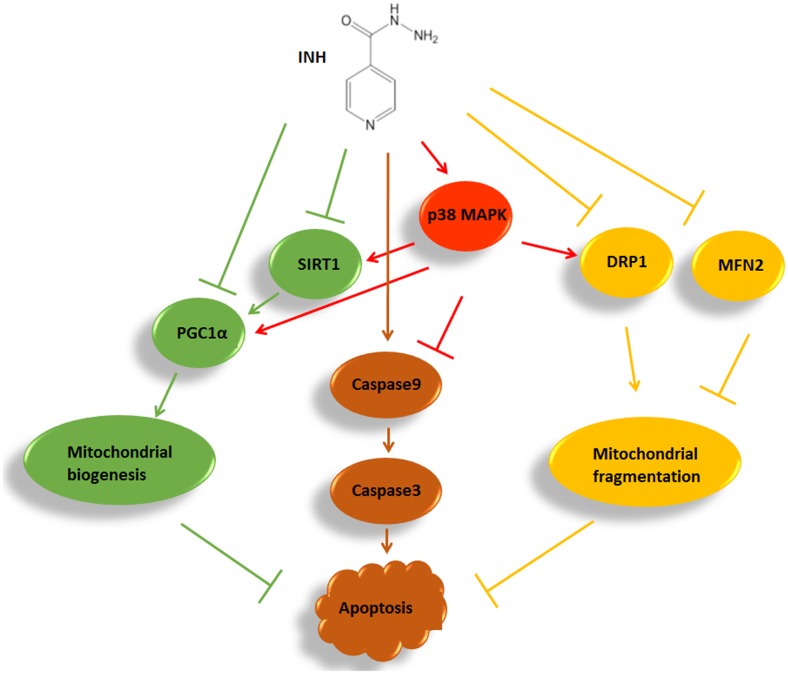
A summary diagram of the pathways involved in INH-induced mitochondrial toxicity. In HepG2 cells, INH inhibited mitochondrial biogenesis, caused mitochondrial fragmentation and exerted apoptotic effects. Meanwhile, INH activated p38 MAPK which not only reduced impairment of mitochondrial biogenesis through partly recovering SIRT1-PGC1α pathway activation, but also promoted protective mitochondrial fission by partial recovery of DRP1 protein expression. Therefore, INH-activated p38 MAPK alleviated apoptotic effects. The effects of INH on SIRT1-PGC1α pathway and DRP1 resulted from the imbalance of many positive and negative roles of upstream pathways. In the study, p38 MAPK was an upstream positive regulator of SIRT1-PGC1α pathway and DRP1. Due to its limited positive effects, p38 MAPK activation failed to completely recover the SIRT1-PGC1α pathway and DRP1 expression. Consequently, although p38 MAPK was activated by INH, the activations of SIRT1-PGC1α pathway and DRP1 were still down-regulated in INH-treated HepG2 cells.

In summary, we identified that INH impaired mitochondrial biogenesis resulting in apoptosis in HepG2 cells by inhibition of SIRT1-PGC1α pathway, and meanwhile induced protective mitochondrial fragmentation against apoptosis involving decreased levels of MFN2 and DRP1. INH activated p38 MAPK, which alleviated apoptosis not only by alleviating mitochondrial biogenesis impairment through partly recovering SIRT1-PGC1α pathway activation, but also by facilitating pro-survival mitochondrial fission via partial recovery of DRP1 expression (**Figure 9**). Therefore, modulation of mitochondrial biogenesis and dynamics by activation of p38 MAPK may provide a potential therapeutic strategy to alleviate INH-induced hepatotoxicity.

## Author Contributions

TZ, LL, RW, XY, and JZ performed the research. TZ, YW, and SP designed the research study. TZ analyzed the data. TZ and TI wrote the paper.

## Conflict of Interest Statement

The authors declare that the research was conducted in the absence of any commercial or financial relationships that could be construed as a potential conflict of interest.

## Abbreviations

c-Cas3: cleaved caspase 3
c-Cas9: cleaved caspase 9
COX IV: cytochrome oxidase subunit IV
DRP1: dynamin-related protein 1
INH: isoniazid
MFN2: mitofusin 2
MMP: mitochondrial membrane potential
NRF1: nuclear respiratory factor 1
PGC1α: peroxisome proliferator-activated receptor γ coactivator 1α
pro-Casp3: procaspase 3
pro-Casp9: procaspase 9
SB: SB203580
SIRT1: silent information regulator two ortholog 1
TEM: transmission electron microscopy
